# Dual-band spectral filter array integrated with a telecentric lens for real-time surface plasmon resonance sensing and imaging

**DOI:** 10.1515/nanoph-2025-0417

**Published:** 2025-12-05

**Authors:** Yi-Hsin Tai, Chih-Hung Kuo, Shenq-Hann Wang, Xiu-Wan Chen, Hsin-Yi Hsieh, Chia-Chun Chang, Pei-Kuen Wei, Chin-Chuan Hsieh

**Affiliations:** 699015 VisEra Technologies Company Limited, Hsinchu 300, Taiwan; Research Center for Applied Sciences, Academia Sinica, Taipei 11529, Taiwan

**Keywords:** DSFA, DBSI, SPRi

## Abstract

Multispectral and hyperspectral imaging have been extensively applied in various imaging domains, where spectral channels with narrow bandwidths provide detailed information for optical signal analysis. The integration of multi-channel filter arrays with image sensors is essential for multispectral detection. To extend this capability to cameras without integrated filters, a dual-band spectral filter array (DSFA) combined with a telecentric lens was employed with a monochrome camera for real-time surface plasmon resonance imaging (SPRi). Placement of the DSFA in front of a broadband light source generated spatially modulated excitation signals incident on a gold-coated periodic silicon nanostructure serving as a surface plasmon resonance (SPR) chip. A pixel-shift-based demosaicing method enabled the separation of checkerboard-like images into two spectral bands corresponding to the filters of the DSFA, facilitating *γ*-based spectral contrast response analysis. This optical configuration successfully demonstrated dynamic monitoring of the interaction between anti-BSA and immobilized BSA on the chip. Compared with wavelength-shift analysis, *γ*-based analysis improved the refractive index detection limit by nearly two orders of magnitude, enabling highly sensitive monitoring of biomolecular interactions. The DSFA-based SPRi platform provides a flexible, highly integrable, and label-free solution for quantitative analysis of biomolecular interactions.

## 1 Introduction

Multispectral and hyperspectral imaging have been extensively explored for applications in fields such as aerospace technology [1], [2], [3], mobile imaging [4], [5], agriculture [6], [7], [8], environmental detection [9], and medical diagnostics [10], [11], [12]. By providing multiple spectral channels with narrow bandwidths, these imaging modalities enable the extraction of detailed spectral signatures that are indispensable for the identification, classification, and quantitative analysis of physical, chemical, and biological properties. Despite their wide utility, the practical implementation of multispectral and hyperspectral imaging often relies on specialized sensors or bulky spectrometers, which increases system complexity and limits broader adoption [13], [14], [15].

To realize multispectral detection, various filter integration strategies have been investigated. Among them, Fabry–Perot (FP) cavity-based filters define spectral channels by precisely controlling cavity thickness, and can be implemented in compact formats on CMOS sensors [16], [17], [18]. However, the relatively broad transition width of FP filters often causes significant spectral crosstalk between adjacent channels [19]. Alternatively, dielectric Bragg reflector (DBR) filters employ alternating multilayer stacks with high and low refractive indices to achieve sharper spectral transitions and narrower bandwidths [20], [21]. Nevertheless, the increased thickness of DBR stacks results in strong angular sensitivity, which further exacerbates spectral crosstalk under non-normal incidence conditions [22].

To overcome these limitations, composite structures that integrate FP cavities with DBR mirrors have been proposed. In such designs, the DBR mirrors act as high-reflectivity boundaries to define the FP cavity, while additional multilayer dielectric band-pass blocking filters are incorporated on the outermost layer to suppress sidebands and further enhance spectral purity [23], [24]. These hybrid architectures can simultaneously improve transmission efficiency, sharpen transition edges, and reduce crosstalk. However, the associated fabrication complexity, thickness control, and integration with CMOS sensors remain challenging, restricting their widespread applicability [25].

In addition, other optical structures such as deep trench isolation (DTI) [26], [27], black matrix shielding [28], [29], metasurfaces [30], and supplementary dielectric layers [31], [32] have been investigated to mitigate angular sensitivity and crosstalk. While effective, these solutions generally involve complex processing or increase system cost, limiting their accessibility for compact, real-time imaging platforms.

Another challenge in multispectral and hyperspectral imaging lies in the requirement for filter wheels [33] or tunable elements such as acousto-optic [34] and liquid crystal filters [35], [36]. While these approaches enable sequential wavelength selection, they introduce switching delays and hinder real-time performance. As a result, most conventional multispectral platforms remain expensive, bulky, or too complex to be easily deployed in applications that demand compact and rapid imaging solutions, such as real-time biosensing.

Surface plasmon resonance (SPR) has been widely applied for label-free biosensing, offering high sensitivity to changes in refractive index at metal–dielectric interfaces. Its imaging counterpart, surface plasmon resonance imaging (SPRi), extends this capability by enabling real-time and spatially resolved monitoring of biomolecular interactions, making it a powerful platform for quantitative biological and medical applications [37], [38], [39].

In this work, a dual-band spectral filter array (DSFA) fabricated at the wafer level was combined with an in-line illuminated telecentric lens to enable dual-channel imaging on a monochrome CMOS camera. Placement of the DSFA in front of a broadband light source produced a checkerboard-like illumination pattern, while the telecentric lens restricted both the incident and collection angles, minimizing angular divergence and improving image stability. The raw checkerboard-like dual-band spectral image (DBSI) was directly captured by the CMOS sensor, containing spatially interleaved signals from the two filters. Through a pixel-shift demosaicing algorithm, the DBSI was separated into two spectral bands, allowing *γ*-based spectral contrast analysis. The proposed configuration demonstrated real-time SPRi of biomolecular interactions, with a limit of detection (LOD) improved by nearly two orders of magnitude compared with conventional wavelength-shift analysis. The DSFA–telecentric lens platform therefore provides a flexible and accessible pathway for extending multispectral capabilities to non-filter cameras, simplifying optical setups, and advancing label-free quantitative biosensing.

## 2 Methods

### 2.1 DSFA fabrication

**Figure 1:**
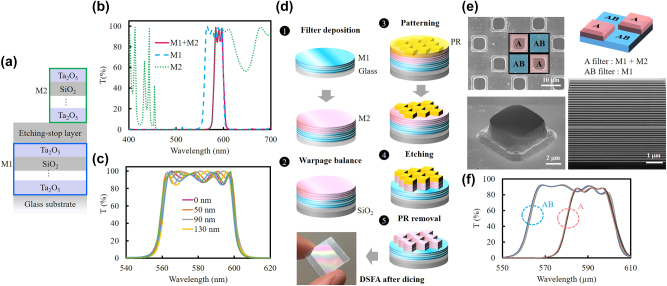
Design, simulation, and fabrication of DSFA. (a) Schematic structures of M1 and the combined structure of M1 and M2. (b) Simulated transmission spectra of M1, M2, and the combined structure (M1+M2) incorporating the SiO_2_ etching-stop layer. (c) Simulated transmission spectra of M1 covered with SiO_2_ layers of various thicknesses acting as the etching-stop layer. (d) Schematic illustration of the DSFA fabrication process. (e) Scanning electron microscope (SEM) and transmission electron microscope (TEM) images of the fabricated DSFA structure. (f) Spectral tolerance within 4 nm, represented by 19 transmission spectra measured at equal intervals on the A and AB filters after deposition of multilayer stacks M1 and M2.

Figure 1(a) illustrates the filter design, which employs two multilayer stacks, M1 and M2, to form dual spectral channels of the filter array. The first channel corresponds to the M1 stack, while the second channel is realized by combining the M1 and M2 stacks. Both stacks are composed of alternating SiO_2_ and Ta_2_O_5_ layers with different thicknesses. To prevent process-induced damage to the M1 stack during fabrication, an SiO_2_ etching-stop layer was introduced between M1 and M2. The simulated transmission spectra obtained using the Macleod for the M1 stack, M2 stack, and the combined structure incorporating the etching-stop layer are shown in Figure 1(b). The spectrum of the combined structure closely corresponds to the combined spectra of M1 and M2, confirming that optical interference between the two stacks is negligible in this design. The simulation results in Figure 1(c) show the transmission spectra of the M1 multilayer stack covered with SiO_2_ layers of varying thicknesses. The results indicate that variations in the SiO_2_ thickness below 220 nm cause almost no spectral shift in the center wavelength of the M1 stack and only slightly affect the transmission intensity. The dual-channel filter array was fabricated on 8-inch wafers, as shown in Figure 1(d). Two filters with distinct spectral channels were realized by depositing multilayer stacks of Ta_2_O_5_ and SiO_2_ with different total thicknesses. The deposition process was divided into two steps. First, an M1 stack with a thickness of 4.5 μm was deposited on a glass substrate to define the first spectral channel in the range of 560–600 nm. To protect M1 from over-etching during subsequent steps, a 200-nm SiO_2_ layer was deposited on the M1 stack as an etching-stop layer. Second, an M2 stack with a thickness of 2.7 μm was then deposited on M1 with the etching-stop layer to create the second spectral channel in the range of 580–600 nm. Mechanical stability of the thick multilayers on the glass substrate was maintained by adjusting the thickness of a SiO_2_ balancing layer deposited on the backside of the substrate.

Individual filter units with dimensions of 10 × 10 μm^2^ were patterned on top of M2 using photolithography. The uncovered regions of M2 were etched to form a checkerboard-like array consisting of two different filters. To optimize the multilayer stack structure, the pattern density of the photoresist is a primary constraint that the etching conditions must accommodate. Therefore, a photoresist-patterned M1 stack was used for the initial test. During the etching process, the flow rates of N_2_, CF_4_, and CHF_3_ were set at 30, 10, and 40 sccm, respectively, and the chamber pressure was stabilized at 5 mTorr. The etching rate was controlled at 1.3 nm/s by adjusting the RF power to 500/85 W. Under this recipe, an etching time of 36 minutes was chosen for the M2 stack. With the protection of the 200-nm SiO_2_ etching-stop layer, an additional buffer etching time of more than 150 seconds was available to prevent over-etching of the M1 stack. After etching, the sidewall angle of the M2 stack was approximately 80°. Finally, the photoresist was removed and the wafer was diced into individual dies. Figure 1(e) shows the top-view, tilt-angle, and cross-sectional images of the DSFA. The transition width in this work was defined as the wavelength span between 50 % and 0.005 % transmission. As shown in Figure 1(f), the cut-on and cut-off steepness of both filters was approximately 4–5 nm. Within their respective passbands, the transmittance ratio exceeded 80 % for the A filter (583–598 nm) and the AB filter (562–598 nm).

### 2.2 SPR chip fabrication

**Figure 2:**
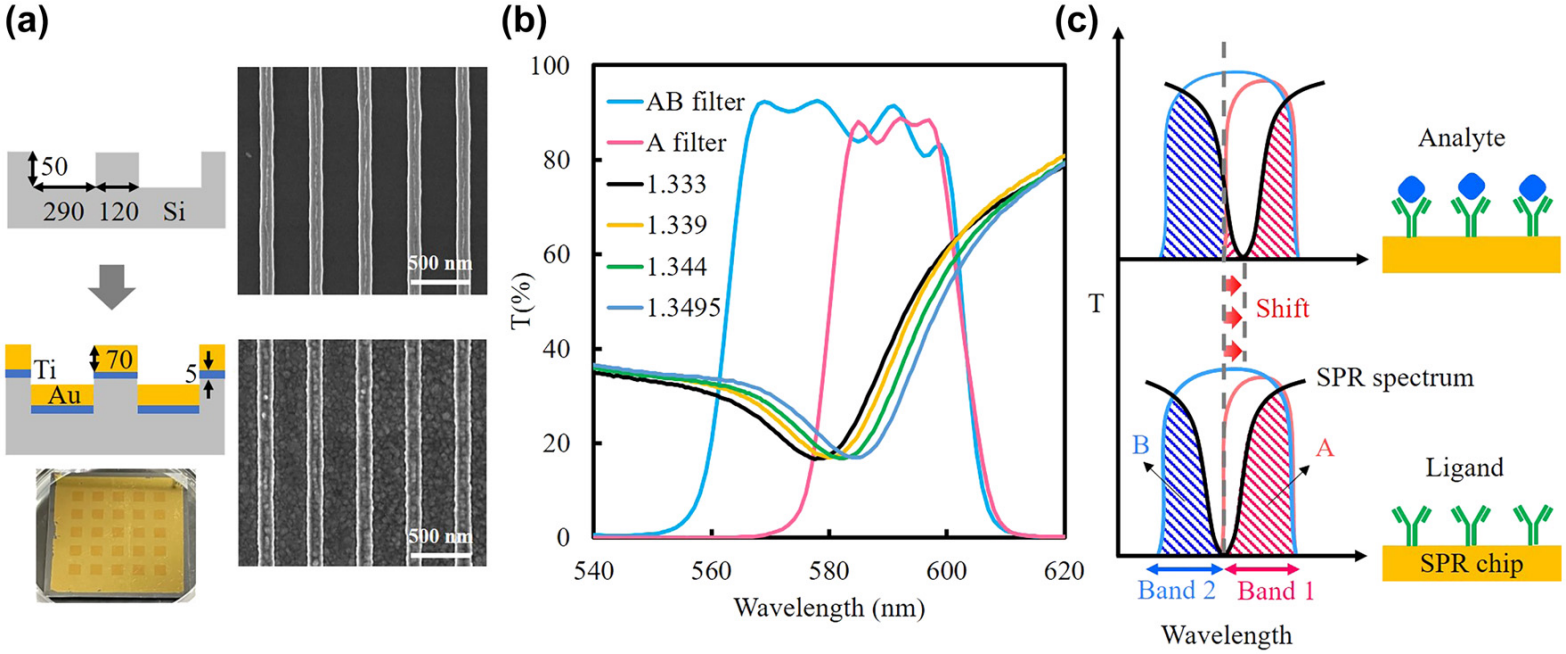
Structure and spectral characteristics of the SPR chip and DSFA spectral alignment. (a) Schematic and SEM images of the SPR chip before and after metal deposition. The inset shows an array of SPR chips with individual dimensions of 5 × 5 mm^2^. (b) Resonance spectral shifts of the SPR chip measured at different refractive indices, along with the transmission spectra of filter A and filter AB of the DSFA. (c) Illustration of intensity variations within spectral band 1 and band 2 during biomolecular interactions between ligand and analyte on the SPR chip.

As shown in Figure 2(a), a silicon grating with a periodicity of 410 nm and a linewidth of 120 nm was fabricated by photolithography and etching. Subsequently, 5 nm of titanium and 70 nm of gold were deposited onto the periodic structure. Scanning electron microscopy (SEM) images of the chip surface before and after metal deposition are presented. For high-throughput measurements, an array of SPR chips was fabricated, with each chip having dimensions of 5 × 5 mm^2^, as shown in the inset [40].

The resonance spectrum exhibited a red shift when the SPR chip was immersed in sugar solutions of varying concentrations, corresponding to refractive indices ranging from 1.333 to 1.3495, as shown in Figure 2(b). To enable DSFA-based detection of spectral shifts, the cut-on wavelength of the A filter and the central wavelength of the AB filter were intentionally aligned with the resonance dip of the SPR spectrum. This configuration divided the resonance into two equal spectral regions, defined as band 1 and band 2, with band 1 corresponding to the passband of the A filter. As illustrated in Figure 2(c), spectral shifts induced by biomolecular interactions on the chip surface were detected by analyzing the intensity variations between band 1 and band 2.

### 2.3 Setup

**Figure 3:**
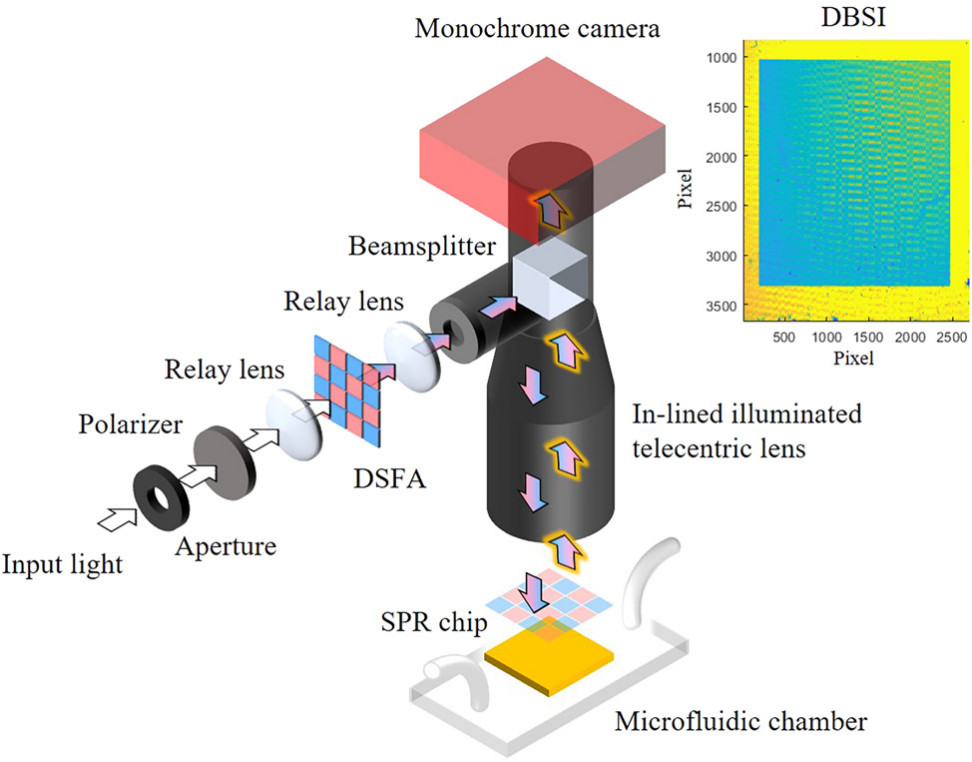
Schematic of the experimental setup integrating the DSFA and a monochrome image sensor. Both the incident angles of the checkerboard-like excitation and the collection angles of the reflected SPR signals are restricted by the telecentric lens.

As shown in Figure 3, the experimental setup consisted of an aperture, a polarizer, a DSFA, and an in-line illuminated telecentric lens (Edmund Optics, 1×, 65 mm WD). A visible light beam was sequentially passed through these components and maintained in TM polarization to excite SPR on the sensing chip. After passing through the DSFA, the polarized beam formed a checkerboard-like array of dual-band spectral channels. This spatially modulated array was directed into the entrance of the telecentric lens by adjusting relay lenses along the optical path. The telecentric lens reflected the checkerboard-like excitation pattern toward the SPR chip via its internal beam splitter, while maintaining nearly parallel illumination and collection paths. This configuration restricted both the incident angles for SPR excitation and the collection angles of the reflected SPR signals, ensuring stable image formation on the monochrome camera (ZWO ASI 183MC Pro), which recorded the DBSI as shown in the inset of Figure 3. The SPR chip was further sealed with a microfluidic chamber to form a flow cell, enabling controlled injection of analyte solutions during the experiments.

### 2.4 Image processing

**Figure 4:**
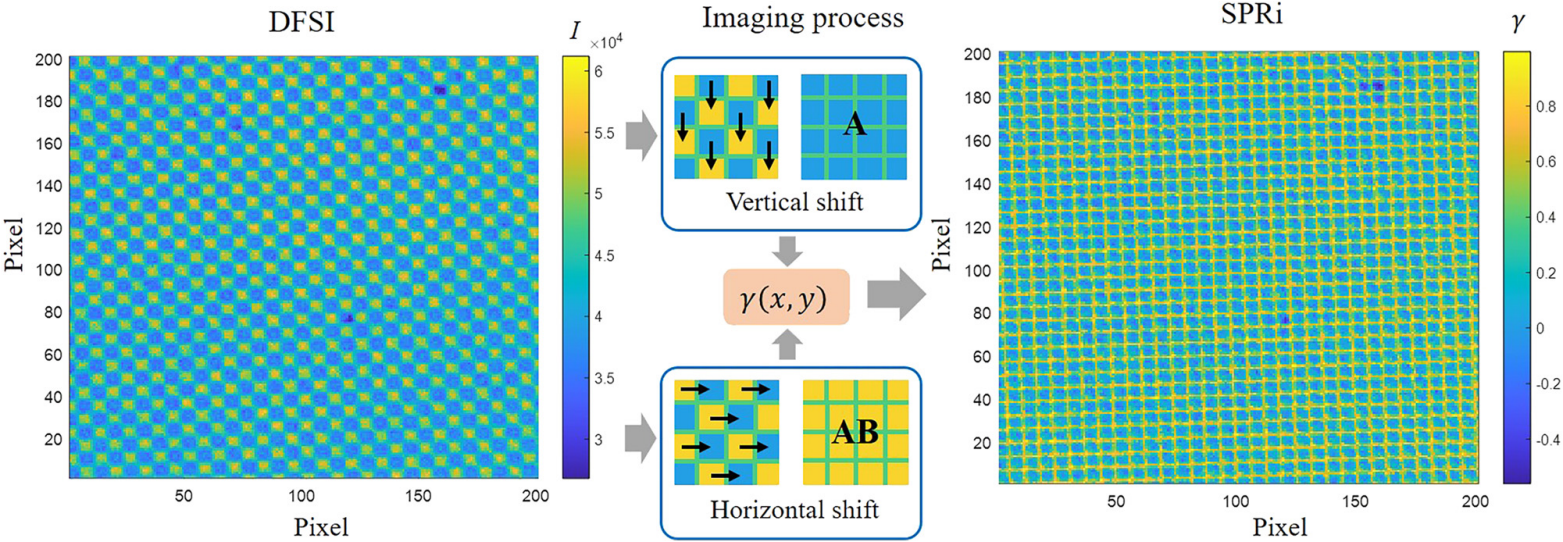
Colorized checkerboard-like DBSI, horizontally and vertically shifted to form demosaiced images for spectral contrast response analysis and reconstruction of the SPRi image.

As shown in Figure 4, the checkerboard-like DBSI exhibited grids of weaker and stronger intensities, corresponding to SPR signals filtered by the A filter with a narrow bandwidth of 583–598 nm and the AB filter with a wide bandwidth of 562–598 nm, respectively. To reconstruct the SPRi image, the DBSI was shifted vertically and horizontally by 10 μm to generate demosaiced A and AB images.

At each pixel position, the spectral contrast response *γ* was calculated to evaluate the relative intensity difference between band 1 and band 2, as illustrated in Figure 2(c). The calculation is expressed as:

$$\begin{array}{ll} \gamma \left(x,y,t\right) & =\frac{I_{A}\left(x,y,t\right)-I_{B}\left(x,y,t\right)}{I_{A}\left(x,y,t\right)+I_{B}\left(x,y,t\right)}\\ & =\frac{I_{A}\left(x,y,t\right)-\left(I_{AB}\left(x,y,t\right)-I_{A}\left(x,y,t\right)\right)}{I_{AB}\left(x,y,t\right)}\\ & =\frac{2I_{A}\left(x,y,t\right)-I_{AB}\left(x,y,t\right)}{I_{AB}\left(x,y,t\right)} \end{array}$$

Where *x* and *y* denote the pixel coordinates, *t* is time, *I*_*A*_ and *I*_*B*_ represent the intensities of band 1 and band 2, respectively, and *I*_*AB*_ is the combined intensity of both bands. Because the passband of the A filter corresponds to half of the AB filter, *I*_*A*_ and *I*_*B*_ remain nearly equal under steady-state SPR conditions when the resonance spectrum is symmetric with respect to the dip, resulting in *γ* ≈ 0. A slight red shift of the spectrum redistributes the intensity, increasing *I*_*A*_ relative to *I*_*B*_ and producing a positive *γ*. Conversely, a slight blue shift decreases *I*_*A*_, resulting in a negative *γ*.

## 3 Results

**Figure 5:**
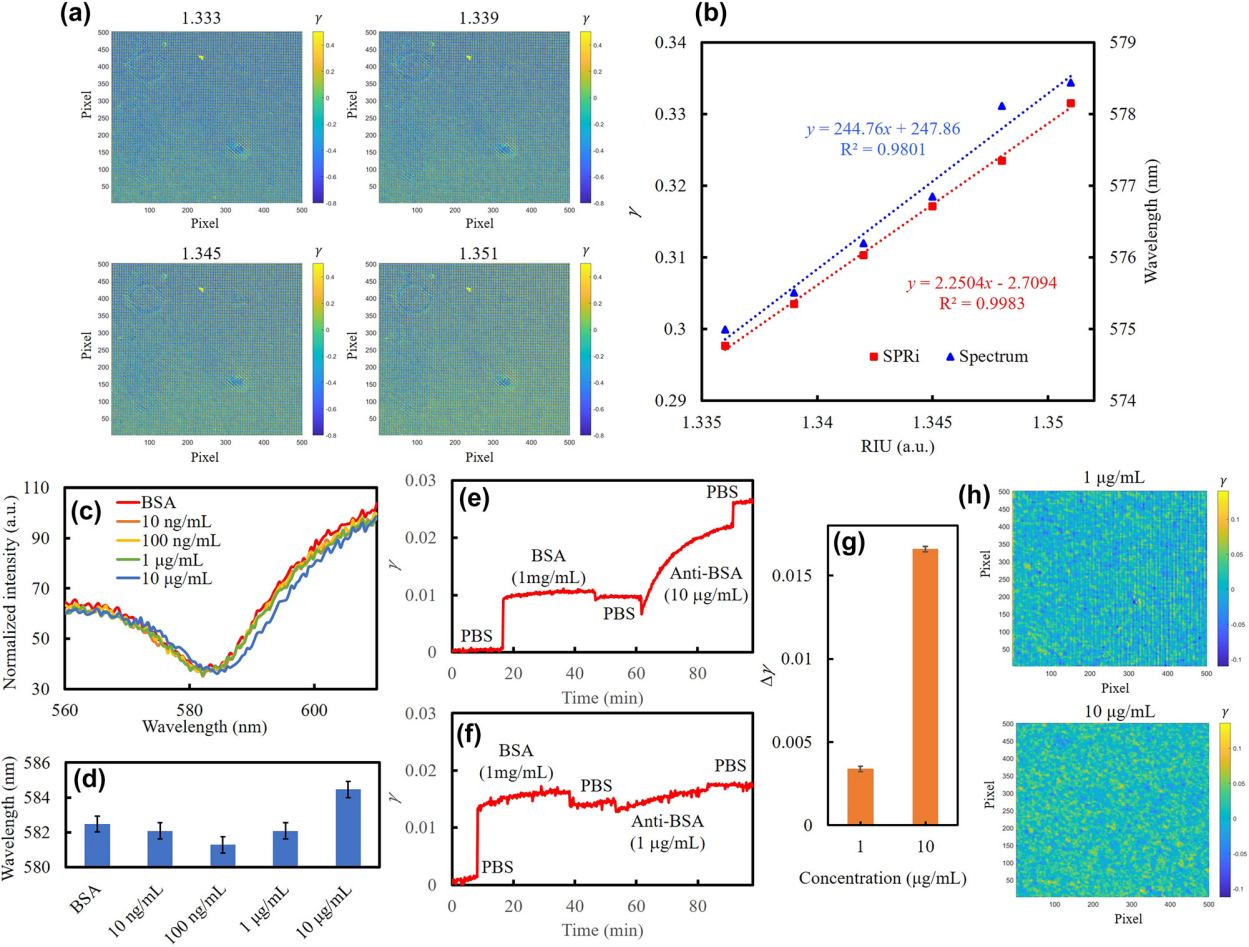
Real-time SPRi and refractive index response using spectral-shift and *γ* analysis. (a) Four SPRi images at refractive indices from 1.333 to 1.351. (b) Comparison of refractive index sensitivities using spectral-shift and *γ* analyses. The corresponding linear fittings as dotted lines; *x* as refractive index and *y* as *γ*, respectively. (c) Spectral shifts during bio-interaction between BSA and anti-BSA. Unlabeled concentrations correspond to anti-BSA. (d) Wavelengths of SPR dips at different interaction steps. Dynamic average *γ* during binding with (e) 10 μg/mL and (f) 1 μg/mL anti-BSA. (g) Bar chart of *γ* differences after PBS washing steps at different anti-BSA concentrations. (h) SPRi differences between the two stabilized PBS states before and after the 1 μg/mL and 10 μg/mL anti-BSA processes.

To evaluate refractive index sensing, sugar solutions with refractive indices ranging from 1.333 to 1.3495 were sequentially introduced into the microfluidic chamber of the SPR chip. As shown in Figure 5(a), the SPRi images reconstructed from the DBSI revealed clear changes in *γ* values with increasing refractive index. The average *γ* values were extracted at each refractive index state and plotted as a function of refractive index. The slope of this dependence defined the refractive index sensitivity of the *γ* analysis shown in Figure 5(b). For comparison, resonance wavelength shifts of the SPR spectrum were recorded using the same optical configuration combined with a spectrometer (BWTEK, Quest™ X). The resonance dip was determined by the minimum transmission intensity within the bandwidth of the AB filter. The sensitivity was determined by linear fitting to be 244.7 nm/RIU for spectral-shift analysis and 2.25 a.u./RIU for *γ* analysis. The errors correspond to the standard deviations of data acquired during 10-minute measurements for each refractive index state, derived from 10 spectra in the spectral-shift experiment and 30 *γ* values in the SPRi experiment. During measurements at different refractive index states, the wavelength error ranged from 0.15 to 0.46 nm, and the error of ranged from 6.80 × 10^−5^ to 1.23 × 10^−4^. The corresponding limits of detection (LOD), derived from the ratio of sensitivity to baseline noise, were calculated as 1.88 × 10^−3^ RIU and 5.46 × 10^−5^ RIU, respectively. These results demonstrate that the *γ* analysis improves the LOD by nearly two orders of magnitude compared with spectral-shift analysis.

For biosensing evaluation, bovine serum albumin (BSA) was immobilized on the gold surface of the SPR chip as the ligand, followed by sequential injection of anti-BSA at concentrations ranging from 1 μg/mL to 10 μg/mL. Each injection was followed by a phosphate-buffered saline (PBS) washing step to minimize nonspecific binding. Resonance spectral dips measured by the spectrometer at each step are shown in Figure 5(c), and the extracted resonance wavelengths are summarized in Figure 5(d). The resonance wavelength stability was 0.46 nm within 10 min of measurement in buffer solution without bio-immobilization, defining the baseline noise. Under these conditions, conventional spectral-shift analysis could clearly distinguish anti-BSA only at 10 μg/mL.

**Figure 6:**
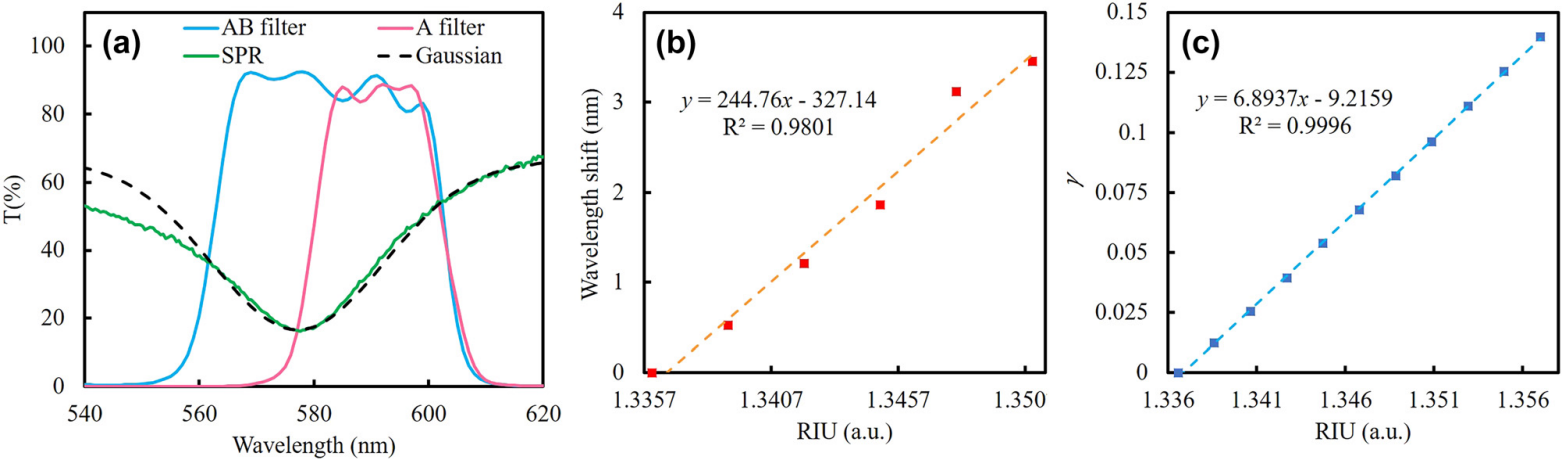
Theoretical sensitivity calculation based on Gaussian-fitted SPR spectra. (a) Average spectra of the two filters, the SPR spectrum, and the corresponding Gaussian fitting curve. (b) Wavelength shift and (c) simulated *γ* variation as functions of refractive index change; dotted-line linear fitting, with *x* as refractive index and *y* as wavelength shift or *γ*, respectively.

In contrast, *γ*-based SPRi analysis exhibited superior stability. The dynamic average *γ* values during the binding processes of 10 μg/mL and 1 μg/mL anti-BSA are shown in Figure 5(e) and (f), respectively. The *γ* differences after PBS washing, summarized in Figure 5(g), confirmed that both 10 μg/mL and 1 μg/mL anti-BSA could be detected. As shown in Figure 4, the grid artifacts on the reconstructed SPRi images, induced by the boundary between the two filters in the DSFA, acted as an interference that reduced the overall contrast of the SPRi. The sensitivity was degraded because the *γ* values associated with these grid artifacts remained unchanged, thereby lowering the change in the average *γ* value. By applying Gaussian smoothing in MATLAB to eliminate the grid artifacts, the contrast of the SPRi images can be improved. Figure 5(h) shows the differences between the two stabilized PBS states before and after the 1 μg/mL and 10 μg/mL anti-BSA processes. By applying Gaussian smoothing, the grid artifacts were eliminated and the contrast of the SPRi images was enhanced. The changes in uniformity on the SPR chip at different concentrations can be clearly observed. To calculate the theoretical sensitivity, the SPR curve was fitted with a Gaussian function to simulate the change in *γ* at different refractive indices, as shown in Figure 6(a). The curves of the A and AB filters were obtained by averaging the corresponding 19 transmission spectra shown in Figure 1(f). Based on the data presented in Figure 5(b), the relationship between the wavelength shift and the refractive index was plotted, as shown in Figure 6(b). According to this fitting function, the change in *γ* during the redshift of the Gaussian function can be converted from wavelength to refractive index as shown in Figure 6(c). The slope indicates that the theoretical sensitivity of the SPRi, 6.89 a.u./RIU, is approximately three times higher than that obtained from the experimental measurement when the interference from grid artifacts is not taken into account. Based on the stability of the *γ* analysis, the concentration LOD of anti-BSA detection was estimated to be as low as 100 ng/mL, underscoring the capability of the DSFA–telecentric lens configuration for high-sensitivity, label-free biosensing.

## 4 Conclusions

Real-time monitoring of interactions between BSA and anti-BSA on an SPR chip was achieved using a monochrome camera combined with DBSI and an in-line illuminated telecentric lens. The DSFA, consisting of two filters with bandwidths of 15 nm and 36 nm fabricated on an 8-inch wafer, enabled reconstruction of DBSI into an SPRi image through *γ* analysis. Compared with wavelength-shift analysis, *γ* analysis improved the LOD of refractive index detection by two orders of magnitude. The DSFA therefore serves as a flexible free-space optical element applicable to any monochrome camera for multispectral imaging. By extending the number of spectral channels in the filter array, the method could be further applied to fluorescence or Raman imaging, enabling dynamic optical signal analysis from a single snapshot.
